# New York Odontological Society

**Published:** 1895-04

**Authors:** 

**Affiliations:** New York Odontological Society


### Reports of Society Meetings.

### NEW YORK ODONTOLOGICAL SOCIETY.

A REGULAR meeting of the New York Odontological Society was held on Tuesday evening, January 15, 1895, at the New York Academy of Medicine, with the President, Dr. Northrop, in the chair.

The minutes of the previous meeting were read and approved.

### INCIDENTS OF PRACTICE AND CASUAL COMMUNICATIONS.

*Dr. J. A. Bishop.*—The plaster cast which I have with me this evening shows the result of a wound caused by a Winchester rifle, the history of which I wish to bring to your notice, as it seems to me of such importance that you may be interested in it for a few minutes.

The patient was a fireman on a through train on the Northern Division of the Missouri, Kansas and Texas Railway, which was held up in December, 1892, by masked robbers. The engineer succeeded in running his train away from them, but the fireman was struck by a rifle-ball, which carried away the lower portion of his face, the lip, alveolus, the teeth, fracturing and taking away part of the jaw, and leaving only one molar tooth on the left side. Here we have a condition of things not easy to overcome.

The patient was taken to a Missouri hospital for treatment, but as far as I could learn no dental surgeon was there.

Had the case received treatment by some one understanding the use of the double splint, the bone would have been supported until it could unite in its place. Then a plate could have been made and inserted, using the one remaining lower tooth as a support, and would thus have become a foundation for further surgical operations.

Instead of proceeding in that way, the method used in this case was to repair the face first, and the drawing together of the parts displaced the fractured bone, and the cicatricial tissue will, I think, prevent any treatment from restoring the bone to its usefulness.

In my paper before this Society, two years ago, it was my wish to have it understood that the great success in the Carleton Burgan case was due to the preparatory treatment by the oral surgeon. This has been an age of improvements and advancement in all branches of science, and the method used in that case was entirely new. The voice was restored, together with the power of receiving and holding food so that the patient could masticate it and perform successfully the necessary deglutition. Then plastic surgery restored the face, and the man is living to-day.

The chief object of this paper is to bring into prominence the supreme importance of having the initial treatment of this kind of injury on the proper lines, so that when the patient is turned over to plastic surgery the conditions of the mouth are healthy and in no way interfere with its success.

The patient to whom we have referred to-night was sent to New York by his local branch of the Brotherhood of Locomotive Firemen, an organization embracing fifty thousand men, nearly every one of whom is constantly exposed to the risk of accident, and the accident is as likely to be about the jaw as elsewhere. Yet, with all these dangers existent, no hospital in this country, that I know of, makes any provision for such cases. In London and Glasgow we find some preparation made for the proper treatment of these injuries; why not in New York? We find, in the Twenty-sixth Annual Report of the Presbyterian Hospital, a list of "Special Consultants," composed of specialists in the different departments of medicine and surgery, and yet no oral surgeon is mentioned. There are fifty-two hospitals of one kind and another in this city, but no one of them makes any provision for accidents to the mouth. I believe that this is chiefly due to the fact that physicians and surgeons have not recognized any dependence on our profession. But I hope that this recognition and co-operation are gradually growing for the good of mankind in general, and that one of their first fruits will be the provision for oral surgery in some of our hospitals.

*Dr. Jarvie.*—A little case occurred to me this afternoon, and on the way here to-night with Dr. Hill I related it to him. Possibly it may give a hint to some one situated as I was this afternoon. There was a young lady, about twenty years of age, at my office with a first superior bicuspid quite badly decayed. After applying the rubber dam and excavating the tooth, I found it very much worse than I had imagined when I commenced. When I had removed all the decay, there was nothing but the inner and outer walls left standing, and they were quite thin. The grinding surface was entirely gone. The young lady was averse to having a crown put on, and nothing but a plastic material could possibly be put in the tooth; and oxyphosphate, in a short time, would wear away and leave the tooth with a ragged and uncared-for appearance. I hardly knew what to do, until it occurred to me that I had a piece of porcelain in the office—several pieces, in fact—that I had for about ten years, and had never used. I took one of them and ground it to fit into the anterior approximal and grinding surfaces, filling up the space almost entirely with the porcelain. I cut a groove in the thickness of the porcelain for the oxyphosphate to work into and hold it, and then pressed it firmly into place. That left the contour of the tooth, as it appeared from the front, very respectable. It made quite a presentable-looking tooth after I got through, and it did not take very long to do it.

*Dr. Meeker.*—I want to ask some advice. I have a patient who has a very bad case of pyorrhœa, from canine to canine. I treated him some time ago, but did not see him for about two months. Three or four days ago he came in, and I looked at his mouth. The central incisor was gone, the lateral was loose, and the gums were all purple; but the peculiar thing was that the upper lip was swollen inordinately. Underneath it was a large mucous patch, probably an inch long. The man said he had not slept that night, and could not sleep without having ice-water to take every few minutes. It seems to me that pyorrhœa had nothing to do with the lips to give them that appearance. I cannot make out what connection the tumefaction of the lips has with the pyorrhœa. If any one can enlighten me, I shall be very glad.

*Dr. Hill.*—How many of his teeth are gone?

*Dr. Meeker.*—One of the centrals.

*Dr. Hill.*—Are the other teeth very loose; all on the upper jaw?

*Dr. Meeker.*—The lateral is quite loose.

*Dr. Hill.*—If you will remember, about ten years ago I had a similar case, and brought the gentleman to this Society. The case was almost identical. The gentleman came to me, and I advised him to have four incisors out; then I told him I would bring him here, and finally I sent him to Dr. Atkinson for treatment. He went there, but got no better. He came back to me, and I extracted those four incisor teeth. Those teeth were as nice and clean as could be, and not a particle of foreign substance was or had ever been on them. I treated the case with sulphuric acid. The singular part of it is, that once a year he appears with some other tooth starting precisely in that way. He is very much frightened, and the moment he feels a little swelling he comes to me. The next tooth that was attacked was the first molar, with a swelling as large as a pea at the margin of the gum. I treated it with sulphuric acid, and cured it. The next tooth was an incisor in the lower jaw, and the bone between the two teeth was entirely gone in two or three days. I cured that too, but now in the lower jaw you can see a space just as if the tooth had been removed. He came to me recently with the second superior bicuspid on the left side discharging pus very freely. I treated him with sulphuric acid. Take an orange-wood stick, sharpen it nicely; then take your knife and roughen it the least bit, enough to hold a dozen fibres of cotton. Insert that up under the gum.

Aromatic sulphuric acid ordinarily is about one to eight. I would try that first, but do not be afraid to use it up to forty per cent. If you think the bone is affected, use the forty per cent. until you are sure you have dissolved all the diseased bone. If that man stays away from me three days after he feels this swelling, it will take me a month to get the best of it; but if he comes immediately, it does not. To show you how quick this last case was, he felt it on Saturday, and he waited until Monday, and as I say, there was a cavity there into which you could put your little finger.

*Dr. Meeker.*—I used sulphuric acid, and I have no objection to taking out all the teeth, for I am sure I cannot control the disease.

I want to control this swelling of the lips. A New York dentist treated the case about five or six years ago.

*Dr. Hill.*—Take out the teeth and use wine of opium plentifully. In this case none of the pulps were dead except one central. Before I extracted I drilled into the teeth to find out.

*Dr. Walker.*—Had the patient of Dr. Hill been under constitutional treatment?

*Dr. Hill.*—Yes; but not the kind of treatment I wanted him to have. He was a homœopath, and took mercury. I did not want him to do that, but he did. He has stopped it now.

Dr. Kirk, of Philadelphia, then read the paper of the evening, entitled "Some Principles relating to Amalgams."

(For Dr. Kirk's paper, see page 214.)

DISCUSSION.

*Dr. Kirk.*—I have demonstrated this to be a fact, and we have another striking illustration of it in the alloys of copper with lead. They are sold in commerce, and known as pot metals. They consist of copper with a large percentage of lead. The resulting alloy, when cold and broken, presents a spongy nidus of copper in which globules of lead appear. Such a combination is in the nature of a solid emulsion. When we produce an amalgam after the manner which has been noted here, we have a similar state of affairs; a mass of amalgam holding in its meshes either unamalgamated filings or portions which are not thoroughly saturated. It is more like a solid emulsion than anything to which I can compare it.

*Dr. Sanger.*—I would like to ask Dr. Kirk if he allows the pellets of gold to remain there after he removes the mercury.

*Dr. Kirk.*—I would remove the gold surface. The object is to get a chemical combination of mercury with the alloy in definite atomic proportions not to form a gold amalgam on the surface of the filling. The sponge gold is used merely to remove the excess of mercury.

*Dr. Clowes.*—I am always interested in the subject of amalgam, but really do not know much about the scientific methods propounded here to-night. I should be at a loss in preparing fillings in that way. I do the thing after the ancient fashion of triturating the mercury and alloy in the palm of my left hand. Neither mortar, nor pestle, nor buckskin, nor chamois, nor pliers enters into my manner of procedure. Considering that I must have mixed fabulous amounts in my open palm, who does not wonder at my survival? I feel thankful every day of my life that we have been blessed with so many good alloys and that they possess so largely the quality of saving teeth. The old reliable Lawrence is almost a standard, and what I said for it many years ago I repeat now, that "with it I have achieved many of the best results of my professional life." It does not occur to me that any one has even thanked me for saying this, although my endorsement could not have been without effect. Under the old *régime* metallic copper was supposed to be a beneficent ingredient of amalgam, but in these later years chemically scientific copper has proved to be a scourge of tremendous proportions. Cadmium in amalgam is considered little better than a "pestilence in the land," and such I proclaimed it in the years gone by before this Society. If in the future scientific research shall bring out better things for amalgam than we now know I will gladly welcome them, but even now in its common-sense form it is the superlative marvel among marvels in our profession.

*Dr. Kirk.*—In writing the paper I endeavored to show that it was the chemical compound of the mercury with the elements of the alloy, which is the only relatively stable portion of the material. It would be impossible to say just what the composition of it is, for it is a compound produced by what we call selective affinity, produced under varying conditions and which would not have the same chemical composition under all circumstances. What we get rid of by squeezing through chamois is all that is over and above the amount of material necessary to produce such a chemical combination. It is a chemical combination that we are trying to arrive at. It is not a danger; it is a hope, and the effort is towards getting rid of that excess of mercury and other metals so we may utilize the remainder which is in chemical combination. That is the essential point of the paper; it is a plea for the rational adoption of a well-known method because it is superior to any other which has yet been offered. It is one that we use empirically, I know, but I tried to bring out the scientific reasons for its accuracy.

*Dr. Bogue.*—Have you ever analyzed the mercury that has oozed through chamois-skin to ascertain if it does carry other metals with it?

*Dr. Kirk.*—It does carry other metals with it. That can be tested by using that excess of mercury in mixing with other filings. An alloy mixed with the expressed mercury will not give a combination at all like the original mixture.

*Dr. B. C. Nash.*—There is a method of absorbing the surplus mercury which has not been mentioned. Several years ago I spoke at the First District Society, giving a method that I believed I was the only one then to use. Dr. Dwinelle had described his method of using freshly-mixed amalgam and driving off some of the mercury by heat, placing that on top of his filling, and in that way getting a harder surface, at the same time absorbing the excess of mercury. I went further than that, and said that I was in the habit, and had been for some years, of using wafers of crystalized amalgam,—taking them on a flat instrument, holding it over an alcohol flame, using judgment in regard to the amount of heat, placing them on top of the freshly-made filling, burnishing into and upon it, and in that way absorbing the excess of mercury. I do not advocate that entire fillings should be made of old, crystallized amalgam, but I do claim that you can absorb the mercury in that way, and, as it is conceded that the other methods that have been described are empirical, that this is as good a way as using gold or any other material for the purpose if you get the result desired. I am satisfied with the method and use it constantly.

*Dr. Bogue.*—I came prepared to find fault with Dr. Kirk, but I cannot. The points that I was going to criticise he himself has taken up and criticised rather more than I would have done. Yet possibly there is a word or two to be said in addition to his able paper, which places us on a track upon which we are not put every day,—things that have been alluded to by the last speaker. If he wants odd ways of absorbing mercury he has only to squeeze copper amalgam and it will absorb quite a bit. I do not understand that to be the question. The essayist has wisely brought before us, not in so many words, the question how best we may adapt a certain filling-material to the conditions which are presented in our daily work. I do not want to take issue with him in one single particular, but I think I may add that I believe Dr. Palmer in the paper which he read here in the latter part of 1894 gave us one *practical* point (when empiricism becomes science I do not know), which was to place a couple of layers of thin tin-foil in the bottom of the cavity which is to be filled with amalgam. He gave us a pretty good reason for it. Perhaps I may diverge here or interpolate a word, and say that he gave us just as good reason for that process as he gave for our not using oxyphosphate of zinc on approximal cavities up under the gum, and lest the gentlemen present shall have forgotten why, I will give the reason: because above the gum line of upper teeth is an antacid condition,—below is perhaps an acid condition. Alkalies destroy the phosphate of zinc; acids do not. He suggested that tin should be put under our amalgam fillings, but in

1873 came the direction to use gold on the surface, to absorb surplus mercury, and I was somewhat ridiculed for the idea.

*Dr. Kirk.*—That tin under amalgam was for a therapeutic effect, was it not?

*Dr. Bogue.*—No, unless you mean that oxidation of the tin is therapeutic. I understood him to mean what you speak of to-night.

*Dr. Kirk.*—I would take exception to that. I believe the lining with tin was for its possible therapeutic effect.

*Dr. Bogue.*—His idea of the introduction of tin was that the tin might oxidize.

*Dr. Kirk.*—My point was the question of the homogeneity of the filling.

*Dr. Bogue.*—That use of gold is not very generally understood. It is not recognized everywhere that gold has a very strong affinity for mercury, and hence it is the best material to use on the surface. It is not recognized that the cohesion of gold with the amalgam filling is very light, and we could not leave much on the surface if we wanted to. It is not recognized that it is the mercury, in many instances, which causes the discoloration of amalgam to which we object so strenuously. Dr. Kirk did not bring that out.

*Dr. Kirk.*—I am not sure that it is, so I did not bring it out.

*Dr. Bogue.*—Many instances can be given where two fillings from the same mix are put in the same mouth at the same sitting; one is a good filling, the other a bad one; one a hard filling, the other a soft one; one an apparently hard filling under the microscope, the other a spongy granular one. What is the reason?

*Dr. Kirk.*—How do you know it is not the silver that is responsible for the discoloration?

*Dr. Bogue.*—We drive off the mercury from one filling by means of pressure and absorption with gold. You put in another amalgam filling by placing it in with no pressure, let it set, having no pressure whatever, and the filling will be black. The first filling will be hard and fine and white in color and accurately adapted to the walls. The other is pretty sure to be granular and not adapted to the wall, and will show a tendency to curl up at the edges.

*Dr. Kirk.*—While that is evidence, it is not necessarily proof that it is due to the mercury. It is due to the mercury plus what it has dissolved in it. What you have in the one filling is a chemical compound, which is the point I am driving at.

*Dr. Bogue.*—I hope you are right, but your contention is hardly proved as yet. It is a novel view of the matter, with a probability that you may be right. Another quality that is constantly alluded to is edge-strength. Incidentally we may infer between the lines that there is edge-strength gained. The matter of edge-strength is one which I think we ought not to consider. That may seem strange, but I should like to recapitulate what I have said in this way: That in this material, which a long while ago Dr. Clowes wanted us to use and we would not, we have a material which, if properly used, may be of infinitely more service than has yet been supposed. We have a material which put in with matrices, malleted in to press the mercury to the surface, and then having the mercury taken off with gold, is admirable. You get a good thing to contour with, and you get a surface which is whiter than a discolored tooth (as a gentleman once said to me). If it is put in with heavy pressure, and the surface be stippled with gold, it will set before the patient leaves the chair, and will stay white if the materials are right. Dr. Kirk, I suppose, left off from his paper purposely, because he had not room enough, the various and diverging effects of gold, palladium, zinc, copper, and aluminum in various dental amalgams. There has been so much said on those subjects by those who have not experimented that I feel a little bit inclined to put a word or two in just here, in view of these constant remarks. It is not, perhaps, generally recognized that from two to five or possibly ten per cent. of gold may make a stronger and whiter amalgam, whereas if you carry it to twenty per cent. your amalgam deteriorates, is softer, and does not set as well. Two or three per cent. of palladium will cause an expansion and whitening of your mass. Carried to excess, glass tubes will break from the expansion. Zinc in small quantity produces a white amalgam, and we all know about copper. I was delighted, when this subject was brought up, at the hit it gives to platinum and aluminum. There are aluminum alloys put on the market when I do not believe it is possible to make an amalgam of aluminum in any way. As it does not amalgamate, it leaves a granular mass perceptible under the finger, and of course it is perceptible at the wall of the cavity.

*Dr. S. G. Perry.*—There is another question in relation to this subject that has not been touched upon, or else I did not hear it. I refer to the spheroidal tendency of amalgam. About ten or twelve years ago Dr. J. Smith Dodge read a paper before this Society, at the house of Dr. Kingsley, I think, on the subject of the spheroidal tendency of amalgam fillings. In that paper he took the ground that amalgam has that spheroidal tendency. I think he expressed what was in the minds of many, and what had been spoken of before, and what had perhaps been accepted as correct; but I do not think the subject had ever been put so clearly or so scientifically. That was the first satisfactory explanation I had for the advantage that I was sure arose from the use of crystal gold for absorbing the excess of mercury. I would not for a moment undertake to discuss this question from the chemical stand-point or from the stand-point of an expert like Dr. Kirk, for we must sit at his feet and learn wisdom as far as those things are concerned. We cannot give the time to those experiments, and we can only view the question from the clinical aspect. I use the amalgam in a moist form first, so I shall be sure to get a perfect adaptation to the walls of the tooth in every way; also to insure a perfect "mixture." Then I get out the excess by using warm instruments and annealed crystal gold. I take out as much as I can, and then finally take off all that is possible from the surface with the crystal gold. I have never found anything else that will compare with it. I have used it for a great many years. I do not know who first used it. It did not originate with me, else I should have remembered my first use of it. As Dr. Bogue has intimated, crystal gold does not adhere, and cannot easily be made to do so, but it will take away the excess of mercury. Dr. Nash's idea I have tried, but I do not think it is as handy or as neat or as satisfactory, and I am willing to pay the extra cost of the gold in that form to be able to take away the excess of mercury in such a quick and satisfactory manner. It has a double effect. It takes away the excess and leaves the filling so hard that if there is a spheroidal tendency there is less opportunity for movement, and the filling does not seem to shrink. The second point is, it leaves a whiter surface. In reference to Dr. Clowes's statement that we are *blessed* with many amalgams, I would say we are *cursed* with many. It has been a strange thing to me that with the means we have, and the earnest desire that exists in the profession, that something has not been done before this in the way of the appointment of a commission or the establishment of some system by which something definite should be known about amalgams, so we should not use everything that is brought to our door. It is a pity that our profession has not settled upon some definite formula from which we may know what to expect. Amalgams have come to stay, although there are still some men who say they have no use in our offices. They have a greater field for usefulness than they ever had. I am sorry Dr. Kirk did not manage to condemn copper amalgam, but he could not do so in his paper, as it did not undertake to discuss different kinds of amalgam. It is the most treacherous filling-material used by our profession.

*Dr. Rhein.*—The essayist in his paper kindly mentioned a method that I advocated in the use of preparing amalgam fillings, some years ago, at a meeting of the New York State Dental Society. In 1880, when I was a student of the late Dr. M. H. Webb, I was almost persuaded at one time to entirely give up the use of amalgam. You all recollect how earnestly he used to talk against the use of it in any shape or form. There never was a man who took a more decided stand against it. He would constantly call attention in his office to the defective results, and to the loss of teeth due to the bad results of fillings, where if proper ones had been inserted the teeth would unquestionably have been saved. At that time my attention was called to the use of gold in finishing off the surfaces of amalgam fillings. Strange as it may seem, Dr. Bonwill was then actively advocating the use of an extra amount of gold, and not bibulous paper, for the best results from amalgam fillings. He had put upon the market about that time an alloy with which a special form of mercury was to be used. This mercury was mixed to a certain extent with gold. In the use of gold to wipe up the excess of mercury and then remove it I did not get a uniformly successful result. The successful cases may have been seventy or ninety out of a hundred, but they were not uniformly successful, such as we would expect to get from gold fillings,—edges that we knew would remain perfect. About 1882 I accidentally hit on a method of inserting amalgam fillings that has given uniformly successful results. By that I mean that after waiting a week or so the amalgam could be finished off properly, and it would remain as perfect and with as bright a color as a gold filling. At that time, while using the Wolrob cylinders, some of them unravelled, and in endeavoring to make use of these for removing excess of mercury in amalgam fillings some of them would disappear under my eyes, and that was the end of them, and I never saw any more of those particular unravelled cylinders. I had tried before that to incorporate gold in the amalgam, but the gold I used was No. 4, and I never was successful in accomplishing it. It would eventually strip. Since then I have used a specially thin gold-foil (about No. 1), placing it upon an annealing lamp and using the proper judgment, so as not to get too large a percentage of the gold in the filling,—a necessity which I have appreciated by experimentation, that too large a mass of gold will unquestionably produce a softer filling. You can not only remove all the excess of mercury in this way, but you can later finish the filling the same as one made of solid gold. In regard to the mass of amalgam that shall be introduced in a cavity, the essayist seemed to object to the operator having a specific weight for the different materials. I think you can obtain a more satisfactory result if, by experimentation having first settled in your mind how plastic you want your mass of amalgam to be, you have reached a definite proportion that the mercury should bear to the alloy. Of course this depends on what kind of a cavity you wish to insert it in. If you have a crown cavity, it is not necessary to have it as plastic as for contouring the side of a tooth. I have a status of figures that suits my convenience, by which I weigh out a certain amount of alloy and a certain amount of mercury. In that way I avoid the necessity of squeezing out any excess of mercury before introducing it. I would present this method to you because some of the gentlemen seemed to imagine that it was impossible to incorporate any of the gold so it will remain. I have never seen any gold that was introduced thin enough at the outset that did not become an incorporated portion of the filling.

*Dr. Palmer.*—In relation to what Dr. Perry said about gold not being incorporated, Dr. Smith has said he has done so frequently. I would like to ask Dr. Kirk what objection there is to allowing it to remain there. Or would it give the atomic result that he wants to reach?

*Dr. Smith.*—Possibly Dr. Palmer misunderstood me. I suggested to Dr. Palmer that I had used the crystal gold, having learned it from Dr. Kirk. It had not occurred to me to remove it after burnishing it on, but it seems to me, from the experience I have, that certainly a good deal of that crystal gold would remain in those fillings. I may be mistaken, but it seems to me to be so.

*Dr. Perry.*—It can be done, I know, with Dr. Klapp's method of filling the lower portion of a large cavity with amalgam, and then topping it with gold. Crystal gold is the only gold by which that can be accomplished. It is true that there can be an incorporation of the gold into the filling, by which a foundation can be established so one can go on after a few layers have been made and build on crystal gold, but I do not use it on the surface of a filling except to get out all the mercury I can. I do not try to incorporate it into the filling, and I think if you undertook on the smooth surface of an amalgam filling to pack on pellets of crystal gold and press them against the filling, you will find they will roll right off. The mercury will unite with the gold and harden it, or if there is not an excess of mercury it will lie there inert and you cannot attach it. I presume Dr. Smith is right when he says you can take very small pieces of gold and rub it in when the filling is fresh.

*Dr. George Allan.*—Do you say you have never used copper amalgam?

*Dr. Perry.*—I have used it, yes; but now I do not use it. I am as prejudiced against it as our friend Dr. McKellops is against ordinary amalgam.

*Dr. Allan.*—Did you ever use copper amalgam as a lining to a filling and put ordinary amalgam over it, so the copper amalgam was not exposed at the edges?

*Dr. Perry.*—No, sir.

*Dr. Allan.*—Would you condemn it?

*Dr. Perry.*—I condemn it as I have used it.

*Dr. Henry Burchard.*—Since hearing the title of his paper I have been grubbing through the literature of metallurgy only to find what a paucity of knowledge of amalgams there is applicable to dentistry. In dental literature itself statements and opinions as to these matters are at present even less generalized and thought over than the stage of hypothesis. Dr. Kirk has clearly shown that much of empiric observation might have been and can be saved were investigators to be governed by the principles of some of the special sciences. Amalgams are usually treated by those who discuss the matter as though this combination of metals were a something *sui generis.* They belong to this general class of alloys as much as any other combinations, and should be so viewed. At a casual glance, one hypothesis seems to be one of glittering possibilities, thus, that the union of mercury with other metals is a fusion, and the setting of the plastic mass a mere physical solidification, due to the combined metals reaching a temperature less than the melting-point.

The one mercury at the temperature of the office is 110° F., removed from its point of solidification, and is molecularly in the condition of any other metal at a temperature one-fifth the way towards volatilization. It would seem, therefore, a safe hypothesis, that in this condition it would act towards other metals like a similar state, so that fusion in it would take place in the direct line of the melting-points of the metals added; thus, the solution of sodium, bismuth, antimony, lead, zinc, aluminum, silver, copper, gold, and then the platinoid metals, would form an ascending scale. Conversely they would solidify, crystallize in the reverse order, and upon once attaining crystallization there would be no further change of form; that is, there will be no spheroiding. Admit the truth of spheroiding, and crystallization cannot have taken place; therefore there has not been a true chemical union between mercury and the alloy.

The fault of this hypothesis, as Dr. Kirk has shown, is that this union is more than a fusion, it is a chemical action, in some cases attended by the evolution of heat and subsequent crystallization, with the union of mercury and alloy metals in definite proportions; it is more than a physical mixture. Also the exceptions, such as aluminum for difficulty of solution and platinum in tardiness of setting, with others that might be named, compel us to a belief in a chemical affinity present.

The subject of washing amalgams has not been touched. The object of this process is the removal of discoloring matter. These are commonly oxides of the metals of the alloy. Should a discolored scum be present in mercury, it is an impurity, usually lead or antimony oxide, not an oxide of mercury, as this metal oxidizes only near its temperature of volatilization. This furnishes an argument for the use of redistilled mercury.

Unless it can be demonstrated that oxides are a desirable addition to an amalgam plug we are compelled to regard them at least as æsthetically undesirable. The only objection to the use of washing with alcohol is the possibility of water remaining in the mass; but as alcohol contains but five per cent. of water, it is questionable if any remains after expressing the surplus mercury. Certainly the washed plug maintains its color better than if unwashed.

A cut in Bödecker's new book shows a section of dentine with the tubuli containing metallic particles for some distance; the pressure used in inserting the plug has caused the escape of mercury and doubtless some of the metals of the alloy in solution into spaces of least resistance. The black metallic salt present here and in other cases must be a sulphide; the sulphur of combination, provided the plug be perfect, can only be derived from decomposing albuminous matter in the dentine. It is this decompositon with the evolution of $H_2S$ which may account for the difference of behavior of two fillings both made from the same amalgam mass; under one there has been decomposition, under the other none. The cut to which reference has been made furnishes an argument for the lining of cavities which are to receive amalgam fillings: preventing the entrance of metals to the tubuli, it prevents discoloration. The other aspect involves the whole question of sterilization and the possible effect of mercury upon protoplasmic filaments.

In conclusion, I believe fully the solution of metals in mercury and subsequent crystallization to be a chemical process, and that Dr. Kirk's argument as to the use of an excess of mercury outweighs those contrary to it; that unless the solvent be present in sufficient amount to insure perfect solution the resulting filling is an indefinite mass in which we cannot accurately determine the effects of action and interaction.

*Dr. Kirk.*—I would like to speak of one point that has been left uncovered, and that is whether we should or should not leave the gold upon the surface of an amalgam filling, and also to speak of Dr. Rhein's recommendation that we weigh the proportions of mercury and alloy. I see no objection to that, but it is quite evident from his statement that in using the weighed proportions he gets an excess of mercury, and that excess of mercury he does get rid of by using gold as an absorbent. Dr. Rhein has made a strong argument in favor of leaving the gold upon the surface of his amalgam fillings. By so doing he has a substratum, or the main body of his filling, composed of a definite chemical compound of mercury with the alloy, and the surface of his filling composed of an amalgam of gold. Is the amalgam of pure gold a desirable thing to have on the surface? Dr. Rhein finds that it is. Then why not make the entire filling of gold amalgam? I have seen nothing yet to shake my faith in the principle which I have tried to bring out in the paper, that we shall take advantage of the selective affinity of the mercury to form definite chemical compounds with the alloys and to use those compounds as the body of the filling, by getting rid of the excess of mercury. I have made the test with respect to leaving sponge gold on the surface of amalgam fillings. It leaves a surface of gold amalgam, which is not as hard, especially on the masticating surfaces, as many of the alloys which we get already prepared for use as amalgams. Therefore I am inclined to think that gold amalgam is lacking in the proper qualities for a good filling. Dr. Hill speaks of washing amalgams. I have used them by washing them, and also without washing them. I see no theoretical objection to washing the amalgam, nor do I see any practical objection, except that it makes a nasty mess and is probably unnecessary. If an alloy is made with an excess of pure mercury, it need not be brought to a fluid state, but to a soft, buttery condition; you can wipe off the soiled mercury on your hand, then after the mass has thickened a little, by putting it into the chamois and squeezing it you get a very satisfactory condition.

*Dr. Hill.*—Are the metals that go out with the mercury the things you want to get rid of?

*Dr. Kirk.*—Yes. Do not understand me as broadly advocating copper amalgam. Copper amalgam is a term that means a great many things. I have in my own mouth copper amalgam fillings put in in 1883, for which I would not take a great many dollars apiece. The subject of copper amalgam is one of the most interesting problems in dental metallurgy. I think under certain conditions there may be a place for copper amalgam. Nobody will dispute the fact that there are some fillings of copper amalgam that have been doing good service and are good fillings now. It is a law of nature that under the same conditions like results flow from like causes, and if it has done so once, copper amalgam will do the same thing again, when we have discovered the factors in its successful use. I have seen good copper amalgam fillings and some abominable ones. We ought to find a place for anything that will help us in the successful adjustment of that series of compromises we are all continually making with dental caries that we call operative dentistry.

*Dr. Perry.*—The fact still remains that the manufacture of copper amalgam at the present time is so uncertain that to make use of it, to commend it, and to favor it, is to put into the hands of many men a very uncertain material, and until the time comes when we can make uniform fillings of it, I say let it alone.

*Dr. Allan.*—The only thing I have to say is to thank Dr. Kirk for drawing our attention so strongly to the fact that it is right and according to correct chemical laws to prepare our amalgams so that there may be a slight excess of mercury, the squeezing out of which carries with it a certain amount of alloy that would be not only useless but even injurious. The mercury unites with definite amounts of alloy to form definite compounds or mixtures. Any excess, therefore, of either mercury or alloy would remain only as an adulteration. The whole question of the combinations of mercury with the metals is very imperfectly understood.

I do not think the essayist would say that we have definite chemical compounds atomically combined, having fixed formulæ in the manner, say, that hydrogen and oxygen, or sodium and oxygen, combine. Again, what conditions or proportions form the hardest amalgams, and how can the contraction or expansion be reduced to a minimum?

*Dr. Kirk.*—My only object in coming here to-night was to bring out the one special point that Dr. Allan has alluded to. Every metal which is added to the amalgam alloys of course confers upon it its own properties, and those properties vary in degree with the amount. As to whether there are chemical compounds formed under the circumstances to which I have alluded, the fact of the formation of a crystalline mass is very strong evidence, if not proof, that it is a chemical combination, and of which a formula might be written out. I gave the classification of alloys suggested by Matthiesson, in which he showed that they were either chemical compounds or solutions or mixtures of any of the three. The compound of mercury with zinc, the compound of mercury with copper or with silver, with gold, tin, and platinum, and a number of others, have all been worked out and found to contain percentages of each of the metals which accorded with the theory of atomic combination. Many of the alloys are simply solutions. The metals dissolve one in the other to an unlimited extent, without any apparent separation point, just as alcohol and water freely mix. In others, if you melt the two metals together and cast them in a cylinder form, you will have them separated in the order of their specific gravity. There is reason to believe that mercury enters not only into the chemical composition, but that it also stands in the same relation to amalgams as water of crystallization does to some salts. The sodium of amalgam is of that class. There is a definite crystalline compound of sodium and amalgam which has the formula $NaH_6$. We probably have a compound in that crystal in which the proportion of sodium to mercury, in the matter of atoms, is two of sodium to one of mercury, and the balance of the mercury affects the crystallization in the same way that water enters into the composition of certain crystals, alum, for example. There are a great many analogies between alloys and the combinations of metals with non-metallic elements. In dental amalgams these chemical compounds of the metals do exist.

One other point in relation to Dr. Allan's question. The order in which these metals are put together does make a difference. We know where platinum is alloyed with tin, we get a definite compound of tin and platinum, and it will be dissolved in the excess of whatever element happens to be associated with it. We generally add a small amount of platinum to a large amount of tin. Therefore we will have a tin compound of platinum dissolved in tin. If we amalgamate that, we will get an amalgam of tin and platinum in combination. Or we may dissolve the platinum in the mercury, and as there has been some question whether that could be done, I will state that I have seen it done, and that it is used for coating steel for certain purposes. Pure platinum amalgam is a soft buttery mass that does not set very well. By mixing together the tin amalgam and platinum amalgam we will get a different combination from that obtained in the first case by amalgamating an alloy of tin and platinum. The order of mixing the alloys is a very important one, and will give different results. I have only mentioned these things. The main feature I wish to bring out is the one central line of thought in the paper, and that is the question of true chemical compounds as the basis for amalgam fillings. The rest would require a volume to illustrate and elucidate.

A unanimous vote of thanks was offered to Dr. Kirk.

Adjourned.

John I. Hart, D.D.S.,
*Editor New York Odontological Society.*

